# Metal contamination at recreational boatyards linked to the use of antifouling paints—investigation of soil and sediment with a field portable XRF

**DOI:** 10.1007/s11356-016-6241-0

**Published:** 2016-02-13

**Authors:** Maria Lagerström, Matz Norling, Britta Eklund

**Affiliations:** Department of Environmental Science and Analytical Chemistry (ACES), Stockholm University, SE-106 91 Stockholm, Sweden

**Keywords:** Antifouling paint, Boatyard, Soil, Sediment, XRF, Cu, Zn, Pb

## Abstract

The application of a field portable X-ray fluorescence spectrometer (FPXRF) to measure Cu, Zn, and Pb in soil and sediments at recreational boatyards by Lake Mälaren in Sweden was investigated. Confirmatory chemical analysis on freeze-dried samples shows that, ex situ, the FPXRF produces definitive level data for Cu and Zn and quantitative screening data for Pb, according to USEPA criteria for data quality. Good agreement was also found between the ex situ measurements and the in situ screening. At each of the two studied boatyards, >40 in situ soil measurements were carried out. Statistical differences in soil concentration based on land use were consequently found: the areas used for boat storage and maintenance were significantly higher in Cu and Zn than the areas used for car parking and transportation. The metal pollution in the boat storage areas is therefore shown to be directly linked to hull maintenance activities during which metal-containing antifouling paint particles are shed, end up on the ground, and consequently pollute the soil. In the boat storage areas, the Cu and Zn concentrations often exceeded the national guideline values for soil. In this study, they were also shown to increase with increasing age of the boatyard operation. Pb soil concentrations were only elevated at a few measurement points, reflecting the phasing out of Pb compounds from antifouling products over the past 2 decades. In the surface sediments, concentrations of Cu and Zn were 2–3 times higher compared to deeper levels. No decrease in metal concentration with time was found in the sediments, indicating that boat owners are not complying with the ban of biocide-containing paints in freshwater introduced over 20 years ago.

## Introduction

Antifouling paints are applied to boat hulls to prevent the adhesion and growth of fouling organisms, such as barnacles. Adverse effects of biofouling include increased fuel consumption, due to increased drag, and loss of maneuverability (Yebra et al. 2004). Antifouling paints containing various types of biocides are frequently used to prevent fouling and, nowadays, Cu is one of the most commonly used biocide (Almeida et al. 2007). Zinc oxide is also readily found in antifouling paints, by itself or in combination with Cu (Watermann et al. 2005).

The use of biocide-containing antifouling paints is an environmental issue as they are toxic to non-target organisms (Karlsson et al. 2010). In harbors and marinas, the leaching of metals from the paints leads to elevated concentrations in the water and accumulation of metals in the sediments (An and Kampbell 2003; Warnken et al. 2004; Schiff et al. 2007; Eklund et al. 2009; Turner 2010; Kylin and Haglund 2010). Recent studies also show that the use of metal-containing antifouling paints contaminates the soil at boatyards (Turner et al. 2009; Eklund et al. 2014). Antifouling paint particles are shed during hull maintenance activities such as water blasting, scraping, and sanding, prior to repainting. Unless collected and disposed of appropriately, the generated paint particles end up on the ground where they can leach metals to runoff and ground water (Jessop and Turner 2011). The spent particles can also be transported to the nearby water and consequently the sediments where they constitute a hazard to deposit-feeding organisms in the benthic environment (Jones and Turner 2010).

There are approximately 880,000 leisure boats in Sweden and, according to a national survey from 2010, 24 % of boat owners use antifouling paints as the principal method to prevent fouling on their boat hull (The Swedish Transport Agency 2010). The survey also showed that 82 % of leisure boat owners do not have access at their boatyards to equipment for proper collection and disposal of shed paint particles. A study at a recreational boatyard in Stockholm inner archipelago has estimated that, based on median concentrations, as much as 2.2 t of Cu and 3.2 t of Zn could be present in the top 20 cm of the soil (Eklund et al. 2014). Out of an estimated 2500 recreational boatyards along the Swedish coast, only 34 have been investigated for soil contamination (Eklund and Eklund 2014). The compilation of the soil data from these 34 investigations revealed high concentrations of metals, often exceeding the national guideline values for soil several fold. Typically, only a few soil samples were collected at each boatyard and the variability in metal concentration was high.

For this study, a handheld field portable X-ray fluorescence spectrometer (FPXRF) was used to measure Cu, Zn, and Pb both in soil and in sediments. The FPXRF allows for non-destructive measurements of a wide range of metals and is commonly used for cost-effective on-site screening of soil at contaminated sites (Kalnicky and Singhvi 2001). The aim of this study was to map the metal concentrations in surface soil at two boatyards to investigate whether differences in land use within the boatyard areas as well as the age of the boatyard could be linked to the degree of soil contamination. Another aim was to evaluate the accuracy of the FPXRF through comparison with chemical analysis. As both boatyards were located by a freshwater lake where the use of biocide-containing paints has been forbidden since 1992 (Swedish National Chemical Inspectorate 1993), a final aim was to investigate the compliance with this legislation.

## Materials and methods

### Study areas

**Fig. 1:**
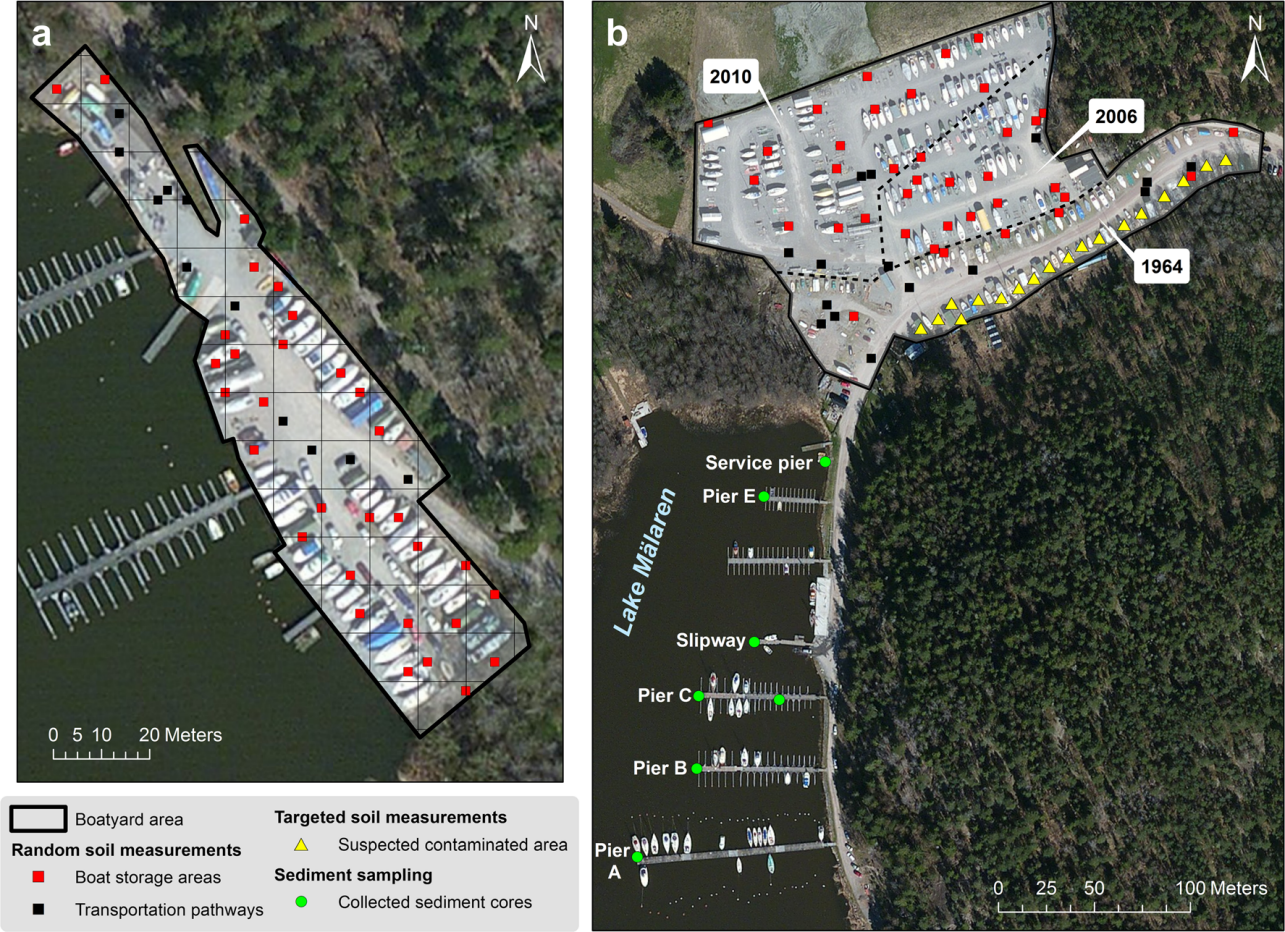
Map of the boatyards *A* (**a**) and *B* (**b**) by lake Mälaren, Sweden. The systematic random soil XRF measurements were performed within a 10-m^2^ grid system (here displayed only for boatyard *A*) within the boatyard area outlined in *black*. Measurement points were classified according to land use: areas used for storage and maintenance of boats (*red squares*) vs areas used for transportation and car parking (*black squares*). For boatyard *B*, areas within the boatyard of the same age are delimited by the *dashed black lines*. The year each area was taken into operation is indicated on the white labels on the map. The underlying satellite images provided through ArcGIS by Bing are from May 2012

The two recreational boatyards investigated in this study were situated by Lake Mälaren, in Sweden (Fig. 1). Both boatyards had been operational since around 1964, were located in the same municipality, and their surfaces were covered with gravel. Boatyard A (Fig. 1a), the smaller of the two boatyards, had an area of approximately 3700 m^2^ and could store around 50 boats in the wintertime. Boatyard B (Fig. 1b) originally had an area of 10,400 m^2^. However, over the years, it had gradually been expanded: 6500 m^2^ was added to the boatyard in 2006 and another 12,700 m^2^ in 2010. At the time of the study (2014), boatyard B stored around 400 boats on its total area of 30,000 m^2^. Sediment samples were collected in the boat harbor by boatyard B where approximately 200 boats were anchored.

### In situ XRF measurements

The equipment used in this study was an Innov-X DELTA-50 FPXRF manufactured by Olympus and equipped with a 4-W, 50-kV X-ray tube. A warm period of 14 days with no rain had preceded the investigation of the boatyard soil and thus the in situ XRF measurements of the soil at both boatyards were performed on dry surface soil. The measurement time was 120 s per point and the soil mode calibration and software using Compton normalization provided with the instrument by the manufacturer was used for quantification. For protection, a piece of 2.5 μm Mylar**®** film was placed across the probe window and the film was replaced between each measurement to avoid cross-contamination. Prior to measurement, debris such as vegetation, pebbles, and rocks were removed and the surface smoothed to allow as much contact as possible between the probe window and the soil. Certified soil standards (NIST 2710a and 2711a) were analyzed every 20 measurements to check the instrument performance. Each time, it was verified that the target elements were within 20 % of the certified concentrations. Typically, the measurements would be within 10 % of the certified values for Cu, Zn, and Pb. A blank sample consisting of clean silicon dioxide (SiO_2_) was also measured every 20 samples to check for potential contamination of the probe window. The limits of detection (LOD) (three times the standard error) were 6, 4, and 3 mg/kg for Cu, Zn, and Pb, respectively. All in situ measurements were >LOD except for two Cu measurements at boatyard A. These were set equal to the LOD (6 mg/kg) for the data analysis.

Measurements at both boatyards were carried out according to a systematic random sampling pattern in a 10-m^2^ grid system (Fig. 1a). The points were located in the field using a GPS with a precision of 2–3 m. The number of sampling points was chosen so that the measurements at each boatyard could be executed within one working day. For boatyard A, one measurement was performed in each 10 × 10 m square yielding a total of 42 measurements. For the much larger boatyard B, 52 10 × 10 m squares were randomly selected for in situ measurement. Eighteen targeted measurements were also performed in the oldest part of this boatyard as there were too few random measurements in this area. Two additional measurements (not shown in Fig. 1) to determine the background concentrations of nearby soils were also made: one on the gravel road leading down to and one in the field North of boatyard B.

In order to assess the land usage impact on the metal concentrations measured in the surface soil, the location of each measurement was classified into two categories: land used for transportation pathways, car parking, etc. and land used for boat maintenance and winter storage. For boatyard B, a distinction was also made based on how many years the areas had been used for boat storage/maintenance as this boatyard had been gradually expanded over time. One-way ANOVA with post hoc testing (Tukey HSD) were performed in JMP® 11.0.0 (*α* = 0.05) for each metal to assess for statistical differences based on land use and age for boatyard B. *t* tests (*α* = 0.05) were otherwise used for comparisons where only two groups were considered.

### Sampling and ex situ measurement of sediments

The sediment samples from marina B were collected in polycarbonate tubes using a Willner core sampler. Seven sediment cores up to 31 cm deep were collected along the different piers (Fig. 1b) and sliced into 3 cm sections on site. All sediment samples were freeze-dried, finely ground in a ball mill, and mixed in order to thoroughly homogenize the samples and reduce any physical matrix effects on the XRF measurements due to variations in particle size (US EPA 2007). The samples were measured with the FPXRF in its bench top stand in low-density polyethylene zip bags with a measurement time of 120 s. Samples were measured three times with repositioning of the bag each time. The average precision ± 1 standard deviation (SD) for the three consecutive measurements of each of the 60 collected samples was for Cu, Zn, and Pb: 7 ± 4, 3 ± 2, and 7 ± 10 %, respectively. The blank sample (clean SiO_2_), the NIST standards, and one of the prepared sediment samples were analyzed every 20 measurements. The measurements of this selected sediment sample yielded a precision of 7, 4, and 6 % (*n* = 10) for Cu, Zn, and Pb, respectively, across the approximately 9 h long measurement session.

### Confirmatory analysis

To assess the accuracy of the XRF measurements, confirmatory chemical analysis by inductively coupled plasma optical emission spectrometry (ICP-OES) was carried out on a subset of the samples. 10 × 10 × 1 cm (w × l × d) soil samples were collected and sieved (<2 mm) at randomly selected in situ measurement points at both boatyards. All samples were freeze-dried and ground prior to both acid digestion and XRF analysis. In total, 21 samples were digested and analyzed: 9 soil samples from boatyard A, 6 soil samples from boatyard B, and 6 randomly chosen sediment samples from marina B.

XRF measurements in the bench top stand were performed using the same procedure as described previously for the sediment samples. The precision for the three consecutive measurements for each of the 21 samples was on average ± 1 SD: 9 ± 7, 9 ± 7, and 8 ± 7 % for Cu, Zn, and Pb, respectively. One of the prepared confirmatory soil samples was also analyzed every 20th measurement in order to determine the precision of the XRF-measurements throughout the whole session (approximately 3 h long). The precision was 6 % for Cu, 8 % for Zn, and 10 % for Pb (*n* = 4).

The 21 confirmatory samples were digested according to the Swedish Standard method SS 028150–2. Briefly, samples and blanks were digested in 7 M nitric acid at 125 °C for 30 min in an autoclave. Analysis was performed on a Thermo Scientific iCAP 6500 Duo ICP-OES with yttrium added on-line as an internal standard in order to correct for drift. Three of the samples were digested in triplicate. A certified reference material for sediment, PACS-3 (National Research Council Canada), was also digested and analyzed in triplicate to verify the accuracy of the procedure. The average recovery ± 1 SD for PACS-3 for Cu, Zn, and Pb was 99 ± 1, 97 ± 1, and 106 ± 2 %, respectively.

In order to validate the accuracy of the XRF method, statistical analyses were performed in JMP® 11.0.0. Prior to analysis, data were log10 transformed to satisfy the assumptions for the use of linear regression as a model. Two main tests were carried out: a slope of “1” and an intercept of “0” were tested for significance at the 5 % level. The results from the statistical tests were used to characterize the data quality level of the ex situ measurements according to the criteria outlined by the US EPA (US EPA 1998) (Table 1). The quality level of the in situ measurements was not classified according to these criteria as these can only provide data at screening level, according to the US EPA (US EPA 2007).

**Table 1:** USEPA criteria for characterizing data quality level (adapted from US EPA 1998)

Data quality level	Statistical parameter
Definitive level	*r* ^2^ = 0.85–1.0 RSD on replicate measurements ≤10 % Inferential statistics indicate the two data sets are statistically similar (test for regression slope = 1 and intercept = 0 at the 0.05 level).
Quantitative screening level	*r* ^2^ = 0.70–1.0 RSD ≤20 % Inferential statistics indicate the two data sets are statistically different.
Qualitative screening	*r* ^2^ < 0.70 RSD >20 % Data has less than 10 % false negative rate.

## Results and discussion

### FPXRF accuracy and precision—ex situ measurements

**Fig. 2:**
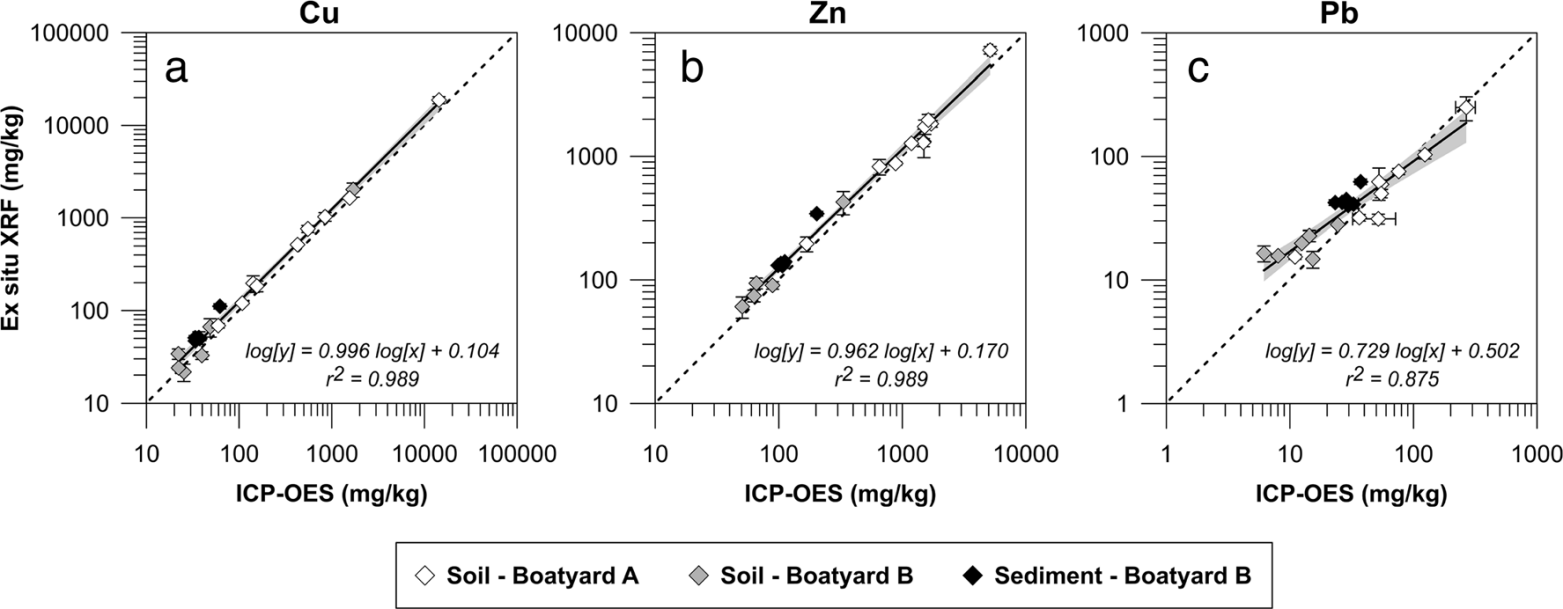
Regression of XRF measurements performed ex situ and ICP-OES results (both in mg/kg dw) from the chemical analyses for Cu (**a**), Zn (**b**), and Pb (**c**). Error bars show the standard deviation of three measurements. The *shaded gray area* represents the 95 % confidence interval of the regression line. The *dashed line* shows the 1:1 correlation

The determined data quality levels for the three metals measured ex situ in the lab stand are shown in Table 2. For all three metals, no differentiation in the FPXRF accuracy based on sample origin was observed (Fig. 2). Excellent coefficients of determination (*r*^2^ = 0.99) were obtained for the regressions with the chemical analysis results for Cu and Zn (Fig. 2a, b) and definitive level data were achieved for both of these metals according to the criteria outlined in Table 1.

**Table 2:** Data quality level for ex situ XRF measurements of Cu, Zn, and Pb determined by comparison with the confirmatory chemical analysis (log10 transformed data)

Element	*r* ^2^	RSD (%)^a^	Slope (95 % CI)	Intercept (95 % CI)	Data quality level
Cu	0.99	9	1.00 (0.95–1.05)	0.10 (−0.01–0.21)	Definitive
Zn	0.99	9	0.96 (0.91–1.01)	0.17 (0.04–0.30)	Definitive
Pb	0.88	8	0.73 (0.60–0.86)	0.50 (0.30–0.71)	Quantitative screening

*CI* confidence interval

^a^Average RSD of all triplicate XRF measurements

**Fig. 3:**
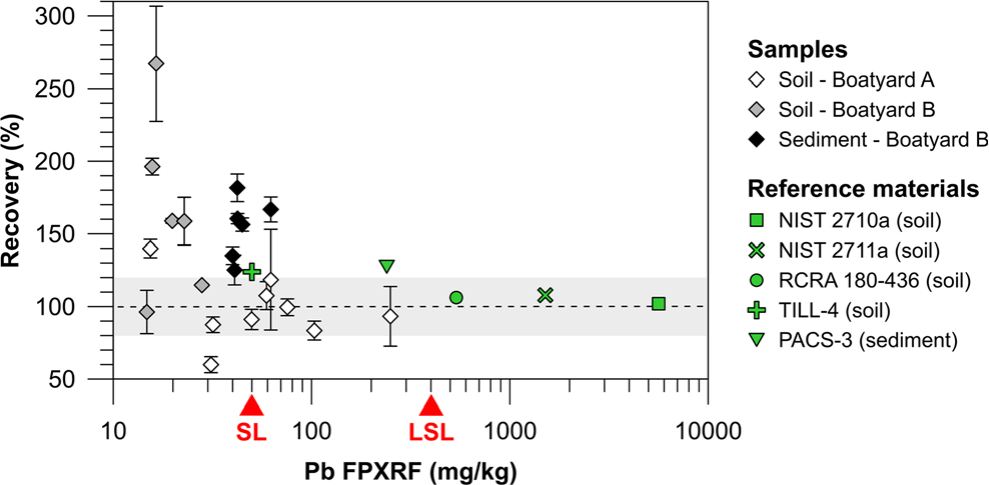
Recovery of the ex situ XRF measurements (white, gray, and black diamonds) compared to ICP-OES values or, for the reference materials (*green symbols*), to certified/recommended values plotted against the concentration measured ex situ by the XRF. The *red triangles* indicate the concentrations of the Swedish guideline values for soil for sensitive land use (*SL*) and less sensitive land use (*LSL*). The *gray area* shows a recovery of 100 ± 20 %

Despite a good linear regression fit between the XRF and ICP-OES measurements (*r*^2^ = 0.88) and an average RSD <10 %, only the criteria for quantitative screening level data in Table 1 could be met for Pb. This is due to the slope and intercept being significantly different from one and zero, respectively. The regression graph for Pb (Fig. 2c) shows that lower concentrations (approximately <75 mg/kg) are overestimated by the XRF which causes the slope to deviate significantly from “1”. A plot of the recovery against the measured XRF concentrations (Fig. 3) shows that about half of the samples exceed a recovery of 120 %. Unfortunately, the randomly selected confirmatory samples only cover a small concentration range for Pb (6–268 mg/kg) and there are few data points in the upper end of this range. Nevertheless, the Pb range covered by the confirmatory samples was representative of the sample set in this study as only 5 % of all the in situ and ex situ measurements exceed it. The recoveries close to 100 % (Fig, 3) obtained for the NIST soil standards 2711a (1400 mg Pb/kg) and 2710a (5520 mg Pb/kg) indicate that the XRF may provide more accurate data at higher Pb concentrations. To assess the XRF accuracy at lower concentrations, closer to those of the samples, additional reference materials were consequently measured: RCRA 180–436 (suggested concentration of 500 ± 100 mg/kg), PACS-3 (certified concentration of 188 ± 7 mg/kg), and TILL-4 (provisional concentration of 50 ± 4 mg/kg). At 106 %, the recovery of RCRA 180–436 was satisfactory. However, for the two lowest standards PACS-3 and TILL-4, recoveries exceeded 120 % at 127 and 124 %, respectively. This finding suggests that at levels below approximately 200 mg/kg, Pb measurements are overestimated due to problems with the internal factory calibration.

The large variation in recoveries and much larger deviations than 127 % for some of the samples indicate however that there may be additional influencing factors. One such factor could be that the acid digestions were not complete as the digestion method employed in this study will not dissolve the refractory metals in the soil and sediment samples. This is probably why most of the Cu and Zn measurements by XRF are slightly higher compared to the ICP-OES results (although this difference is not significant). Even though the Pb recovery for PACS-3 showed good results, an underestimation due to incomplete digestion cannot be ruled out. Another factor may also be particle size. Despite the samples being finely ground, the particle size of the samples will not be completely homogenous and may not match that of the reference standards that were initially used to calibrate the instrument. Kilbride et al. (2006) investigated the effect of particle size distribution on their soil measurements but found no effect for Cu, Fe, Pb, or Zn for either a dual source or an X-ray tube FPXRF. Yet another factor which could have influenced the Pb results is the occurrence of interferences. Gutiérrez-Ginés et al. (2013) also reported inaccuracy for Pb at lower concentrations. In their discussion, they mention the possibility of interference from As. If the Pb Lα line is used for FPXRF quantification, spectral overlap between the Pb Lα (10.55 kev) and As Kα (10.53 kev) peaks can result in inaccurate results: at equal Pb and As concentrations, the As Kα peak will have three times the intensity of the Pb Lα peak (Revenko 2002). To avoid this interference, the soil calibration provided with the FPXRF used in this study was made for the Pb Lβ line (12.61 kev) which is set on the instrument to be detected between 10.40 and 12.80 kev. For this line, interferences from the following elements are nevertheless possible: Se Kβ (12.50 kev), Kr Kα (12.65 kev), and Ac Lα (12.65 kev). However, these elements have a low abundance in the crust compared to Pb (Haynes 2014) and are therefore not very likely to interfere. Another possible interfering element is Fe through the occurrence of an Fe sum peak. Sum peaks can occur when two identical photons enter the detector nearly simultaneously (Bertin 1975). In the case of the Fe sum peak, the detector accidentally counts two Fe Kα photons at 6.40 kev as one, yielding a signal at 12.80 kev (Prange et al. 1993). This potential interference could perhaps explain the bias towards higher concentrations that is visible for some of the lower samples.

Regardless of the cause, the overestimation of low Pb samples puts constraints on how and if the data can be used. There are two national guideline values for pollutants in soil in Sweden dependent on land use: one for less sensitive land use (LSL) and one for sensitive land use (SL). The former is intended for industrial areas whereas the latter is for non-industrial areas such as residential areas. The guideline values were set to protect both the health of people visiting/residing in the area as well as the soil and water environment but are not legally binding (Swedish EPA 2009). Fortunately, the Swedish LSL guideline value for Pb of 400 mg/kg is in what seems to be the more accurate range of the FPXRF. The same cannot be said for the SL guideline value of 50 mg/kg which means that a risk classification of soil at the studied sites relative to this lower guideline value will be more uncertain as XRF values close to the SL value may be overestimated. For the sediments, almost all measurements were ≤75 mg/kg which makes data too uncertain to present and discuss.

### Representativity of field measurements—in situ measurements

**Fig. 4:**
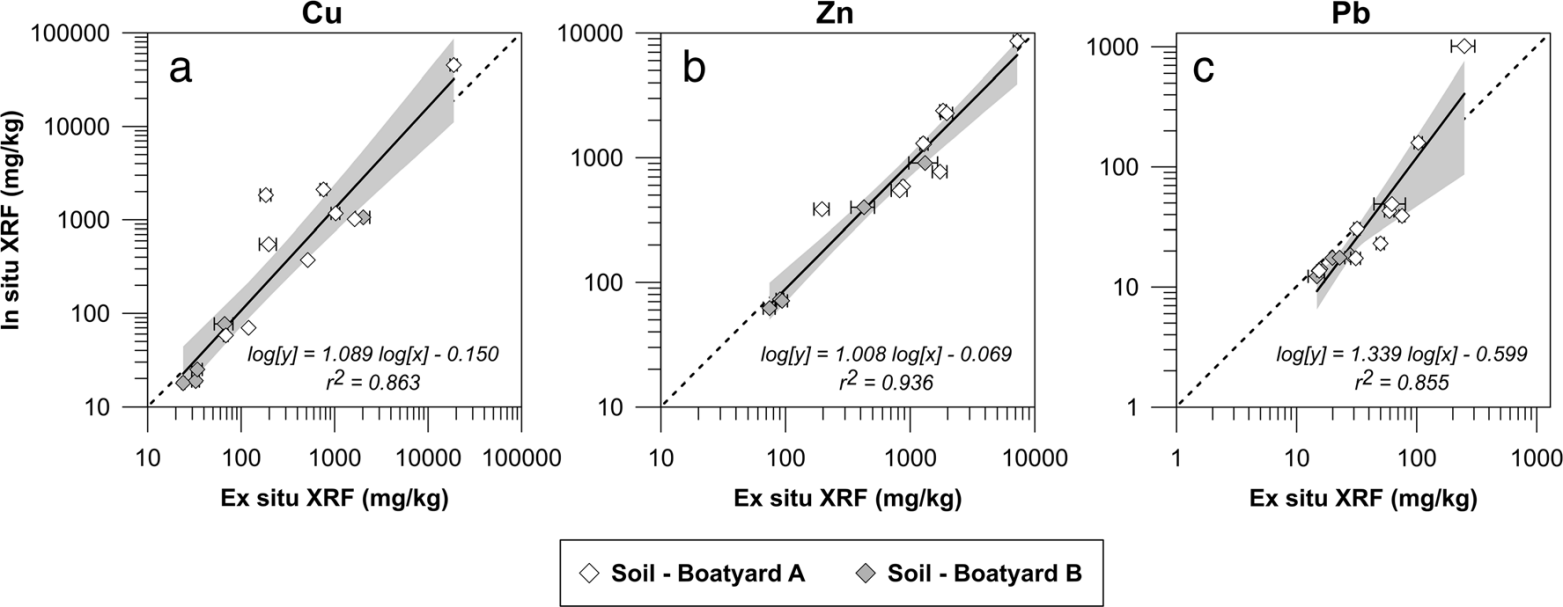
Regression of XRF measurements (mg/kg dw) performed in situ and ex situ. Cu (a), Zn (b), and Pb (c). Error bars show the standard deviation of three measurements. The *shaded gray area* represents the 95 % confidence interval of the regression line. The *dashed line* shows the 1:1 correlation

The coefficients of determinations between in situ and ex situ XRF-measurements (Fig. 4) for all three metals are high (*r*^2^ > 0.85). The regression slopes are close to “1” for Cu and Zn whereas for Pb, the highest data point causes the slope to deviate significantly from “1”. If this data point is removed, the regression equation becomes Log[y] = 0.980 Log[x]–0.083. Despite the good linear fit, several data points fall outside the 95 % confidence interval and the data points are more scattered than for the regression graphs between the ex situ XRF measurements and the results from the chemical analyses (Fig. 2).

There are several parameters which can affect the quality of the XRF measurements in situ. Soil moisture is one such parameter, where a high water content leads to a decrease in the measured concentration by the XRF as water absorbs the characteristic X-rays of the analytes (Ge et al. 2005). It has previously been reported that the moisture content is not a major source of error if it is below 20 % (US EPA 2007; Potts and West 2008). A recent study revealed however a 1.15–1.75 % loss in analyte concentration per every 1 % increase in moisture (Parsons et al. 2013). As all the in situ measurements in this study were carried out during dry soil conditions (i.e. no rain for several days before the measurements), moisture content would only be able to explain a minor portion of the observed scatter. Another factor that could account for the discrepancies between the in situ and ex situ measurements is differences in particle size distribution which may bias the in situ determinations due to their heterogeneity (US EPA 2007). The samples measured ex situ were passed through a 2-mm sieve and finely ground before analysis, reducing surface irregularities and particle size in addition to homogenizing the sample.

As only an area of approximately 0.50 cm^2^ was measured in situ with the FPXRF whereas the ex situ measurements were made on 100 cm^2^ samples, a perfect fit between the two is difficult to achieve, especially considering the uneven distribution of paint flakes on the soil at a boatyard. However, based on the good regression fits obtained, the individual XRF determinations can be considered to be representative of the collected 1 cm deep bulk samples. The in situ measurements in this investigation with this instrument can therefore be used for an accurate analysis of the contamination situation.

### Metal contamination at the recreational boatyards

#### Soil (in situ)—boatyards A and B

**Fig. 5:**
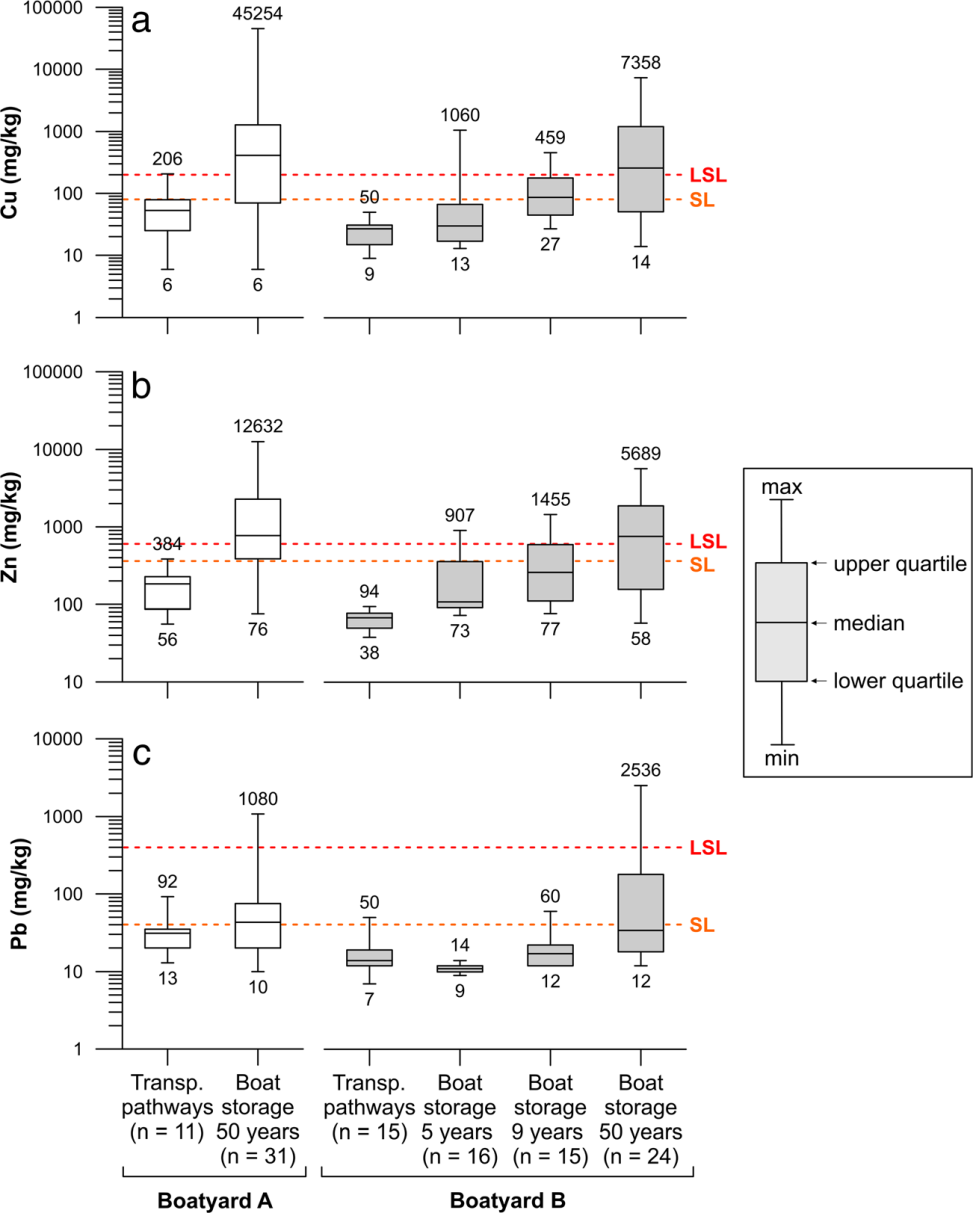
Cu (**a**), Zn (**b**), and Pb (**c**) results from the in situ soil measurements carried out with the XRF at the two boatyards. Data have been sorted based on land usage and also age of operation at the time of the study. The *dashed orange* and *red lines* indicate the Swedish guideline values for soil for sensitive (SL) and less sensitive land (LSL) use, respectively

High in situ concentrations of Cu and Zn in the 1000–50,000 mg/kg range were commonly found in the boat storage areas at both boatyards (Fig. 5). The measured Pb concentrations in these areas were generally at least an order of magnitude lower than Cu and Zn. This is consistent with the current composition of antifouling paints: Cu and Zn are frequent ingredients in contemporary antifouling paints whereas Pb has more or less been phased out over the past 20 years. Since the total ban of TBT (tributyltin) in the EU in 2003, Cu has been the main biocide added to antifouling paints (Yebra et al. 2004; Almeida et al. 2007). Zinc oxide is not listed as an active substance in antifouling paints under the Biocidal Products Regulation (528/2012) or in the preceding European Union’s Biocidal Products Directive (98/8/EC), but is a water soluble pigment added to Cu-based paints as a booster biocide and for its ability to enhance the erosion process of the coating (Watermann et al. 2005; Yebra et al. 2006). Red lead (Pb_3_O_4_), however, was prohibited in consumer products such as antifouling paints in 1995 and the use of other lead compounds such as lead white (Pb_3_(OH)_2_CO_3_) and lead chromate (PbCrO_4_) as pigments in paint has substantially decreased over the past 2 decades (Swedish National Chemical Inspectorate 2007; Turner 2014). This is reflected in the findings of this study: no data points in the newer areas of boatyard B (5 and 9 years old) taken into use after the ban of red lead in antifouling paints exceeded the higher soil guideline value. Pb has a strong affinity to bind to organic matter in the soil and the ability to out-compete other metals for binding sites (Baker 2008) which could explain why it is still occasionally detected in the topsoil layer in the 50-year-old boat areas at both boatyards, despite the 20-year-old red lead ban. Maintenance of boat hulls with historic coatings containing Pb could be another explanation. Some boats may also have lead keels which could potentially act as sources of Pb to the environment. However, in 2005, it was estimated that only 500 sailing boats with lead keels were sold in Sweden per year. Furthermore, the release of Pb from the keels has been deemed as low due to the slow corrosion factor and the fact that the keels tend to be covered by either antifouling paint or plastic hulls which act as protective barriers, reducing the risk of dispersion of Pb into the environment (Swedish National Chemical Inspectorate 2007).

**Fig. 6:**
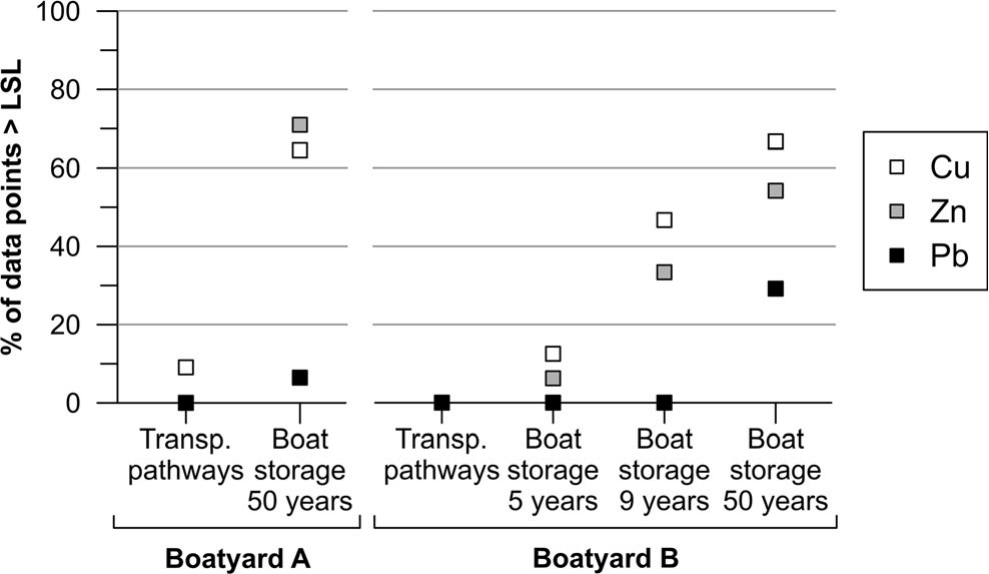
Percentage of the data points within each boatyard that exceeds the Swedish guideline value for less sensitive land use (LSL)

For boatyard A, a statistically significant difference between the two types of land use (transportation pathways/parking versus boat storage/maintenance areas) was established for Cu and Zn (*p* < 0.0001 for both metals). For Pb, however, land use differences had no apparent effect on soil concentrations (*p* = 0.1168) which were, in general, low for both categories of land use types most likely due to reasons already discussed. Only 2 out of 31 points in the boat storage area showed Pb concentrations above the LSL guideline value of 400 mg/kg. This can be compared to Cu and Zn at the same boatyard for which correspondingly 65 and 71 % of the data points in this category exceeded the respective higher guideline values of 200 and 500 mg/kg (Fig. 6).

For boatyard B, statistical analysis showed similar results as for boatyard A: the 50-year-old boat storage area had significantly higher levels compared to the transportation pathways for all three metals (*p* ≤ 0.0002 for all). At boatyard B, two measurements were made to determine background concentrations of the nearby soils. The concentrations of both the soil of the gravel road leading to the boatyard (25 mg Cu/kg, 101 mg Zn/kg, and 23 mg Pb/kg) and the one in the field just North of boatyard B (34 mg Cu/kg, 104 mg Zn/kg, and 38 mg Pb/kg) were low and comparable to the median concentrations of the transportation pathways (27 mg Cu/kg, 68 mg Zn/kg, and 14 mg Pb/kg). Furthermore, the median concentrations measured on the transportation pathway areas were below the SL and LSL guideline values for all three metals at both boatyards. These results indicate clearly that soil contamination by these metals in the boat storage areas is a consequence of boat maintenance such as hull sanding and pressure hosing performed. Statistically higher concentrations of Cu and Zn compared to the transportation pathways were also detected in the boat storage area used for only 9 years (*p* = 0.0233 for Cu and *p* = 0.0017 for Zn). However, for the 5-year-old boat storage area, no significant difference compared to the transportation pathways was detected (*p* = 0.6020 and *p* = 0.1083 for Cu and Zn, respectively). For Pb, only the 50-year-old area is significantly higher compared to the roads (*p* = 0.0002). It should be noted that any spread of contaminants from the 1964 area to the younger areas at this site through transportation with runoff can be ruled out due to the area’s topography: the boatyard slopes downwards towards the bay with a height difference of 10–15 m between the northern most corner of the boatyard and the shore. Hence, it seems that boat hull maintenance carried out on unprotected soil merely for a few years (between 5 and 9 years) is enough to result in a noticeable increase of Cu and Zn concentrations in soil. Figure 6 reflects this finding: there is a clear increase in the number of the data points that exceed the higher guideline value for soil (LSL) the longer the area has been used for boat storage and maintenance. Comparing the 50-year-old boat storage areas in the two boatyards A and B to each other shows that they are equally polluted as there is no statistical difference between them for any of the metals (*p* > 0.2 for all).

The total content of Cu and Zn in the surface soil (top 1 cm) was calculated by determining the areas of the land occupied by the two different types of land use. For boatyard B, the surface areas of the boat storage areas of different ages were also computed. The corresponding median concentration of the metals was used to calculate the total amount of metals in the topsoil within each area. Soil bulk densities typically range between 1.1 and 1.8 kg/m^3^, with lower densities for soils rich in organic matter and higher densities for sandy soils (McKenzie et al. 2002). As the soil at the boatyards was not perceived to have especially high amounts of organic matter but to be more towards the sandy type, a density of 1.5 kg/m^3^ was assumed. The spatial analysis showed that 72 % of the area at boatyard A is designated for boat storage/maintenance. The equivalent number for boatyard B is 67 %. For boatyard A, it was calculated that there is a total of 19 kg Cu and 36 kg Zn in the surface soil. Of these total amounts of Cu and Zn, the boat storage areas account for 95 and 92 %, respectively. For the larger boatyard B, the total amounts were 58 kg Cu and 172 kg Zn in the top 1 cm layer of the soil. Again, the boat storage areas account for most of the total amounts of Cu (93 %) and Zn (94 %). Around 2/3 of the total amounts of the two metals at boatyard B are located in the 50-year-old boat storage area, even though it only accounts for 22 % of the total area. As concentrations in the deeper soil are probably also elevated, especially in the oldest boat storage areas, it is likely that the soils at these boatyards hold large reserves of metals.

#### Sediments (ex situ)—site B

**Fig. 7:**
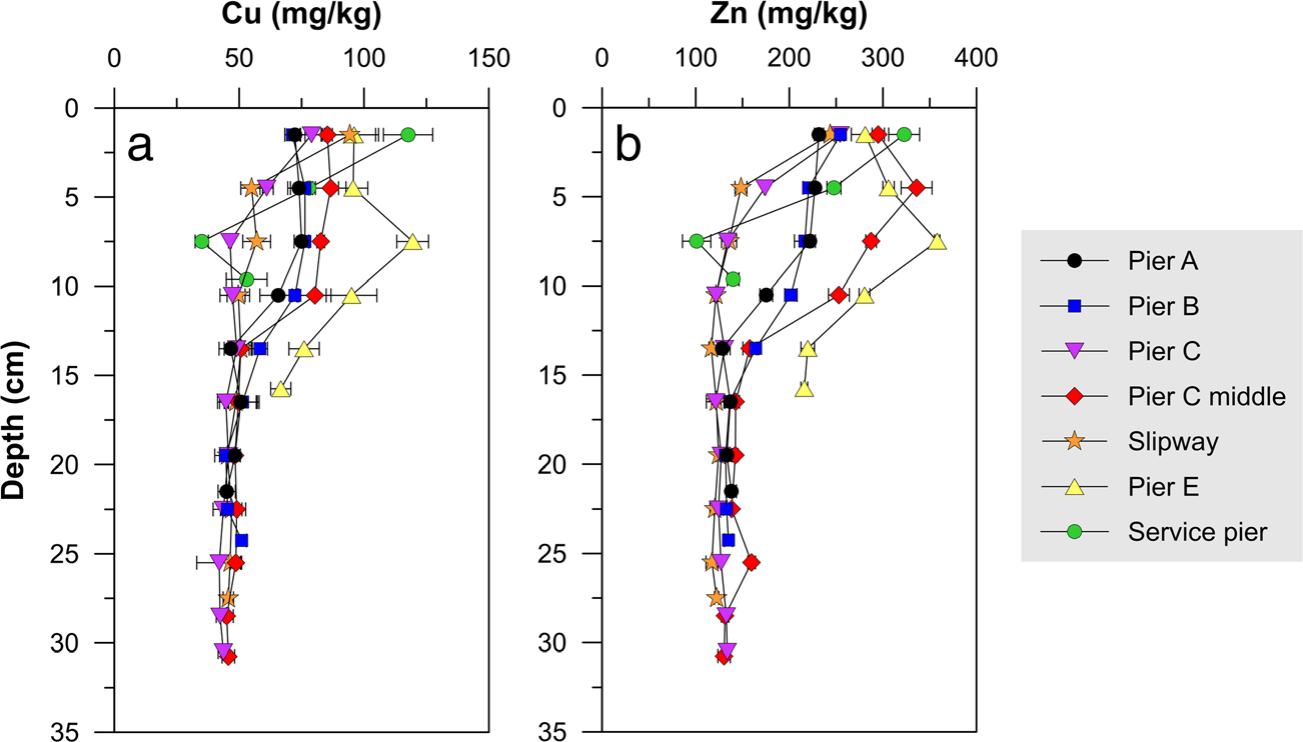
Sediment depth profiles for Cu (**a**) and Zn (**b**) for the seven collected cores collected in the harbor at boatyard B, as determined through ex situ FPXRF analysis. Error bars show the standard deviation of triplicate measurements

The distributions of Cu and Zn in the sediments display the same vertical trend: low concentrations at depth and an increase towards the surface (Fig. 7). Below 17 cm, the profiles converge around 45 mg/kg dw for Cu and 130 mg/kg dw for Zn. No dating of the sediments was carried out in this study but the high degree of convergence at depth would suggest that these could be background values. A survey was carried out by an environmental consultancy company in 2010, in preparation of dredging activities between the slipway and Pier E. The concentrations in two sediment samples at depths between 40 and 60 cm were analyzed and were 31.6 and 24.6 mg/kg for Cu and 93.9 and 82.5 mg/kg for Zn (Ramböll 2010). A study was also carried out in Västeråsfjärden in 2014, located in the western part of Lake Mälaren, to determine the background concentrations in the sediments. This study concluded that the background concentrations were 26 mg/kg for Cu and 134 mg/kg for Zn (JP Sedimentkonsult AB 2014). The concentrations in both mentioned surveys are similar to the <17 cm concentrations measured in this study, suggesting that they are background concentrations.

**Fig. 8:**
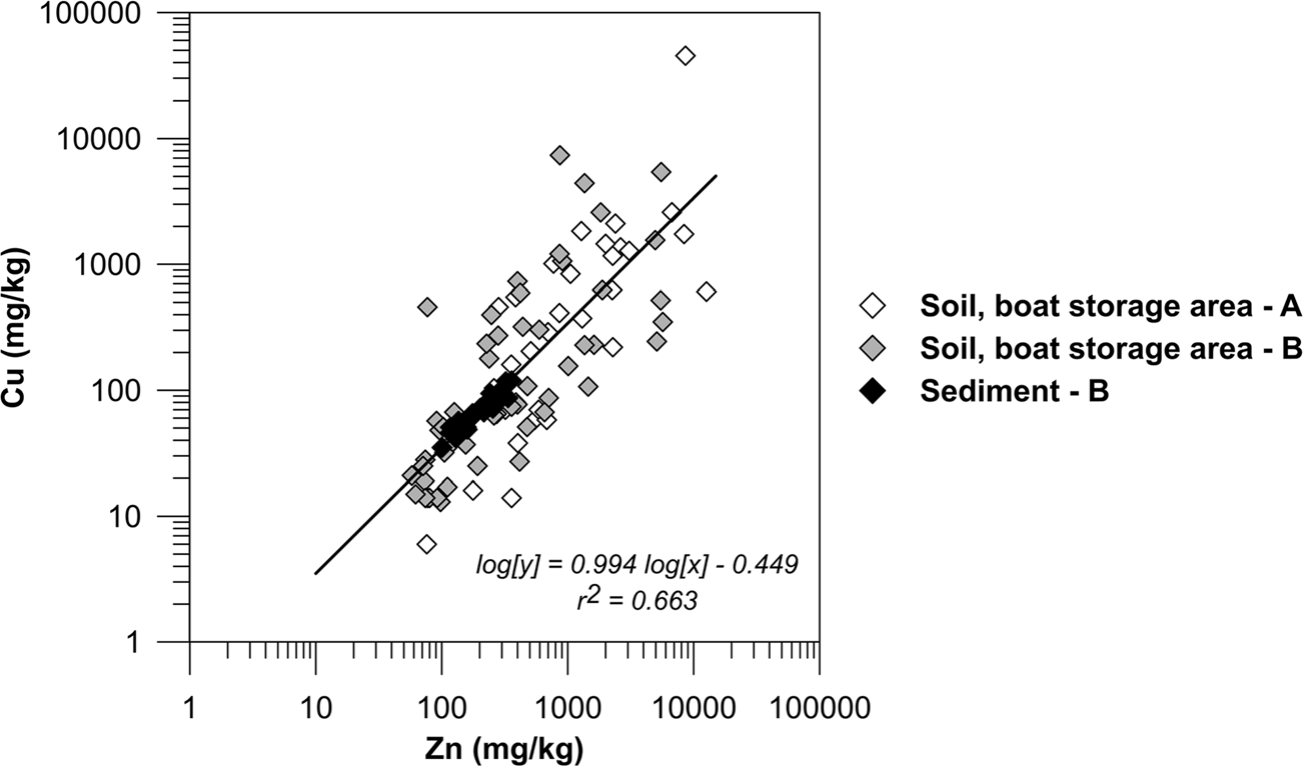
Correlation between Cu and Zn concentrations in soil and sediments

Cu and Zn concentrations at the sediment surface are between 2 and 3 times higher than the levels <17 cm depth. According to the sediment classification system suggested by the Swedish Environmental Protection Agency consisting of five categories, these levels (75–125 mg Cu/kg dw and 225–350 mg Zn/kg dw) are classified as category 4/“high” or category 5/“very high” (Swedish EPA 1999). The increase in concentration for sediments >17 cm is most likely caused by the leaching of Cu and Zn from antifouling paints on boat hulls and/or paint particles in the sediments. The concentration of the two metals was found to be highly correlated in the sediments (*r*^2^ = 0.93). The linear fit obtained suggests that Cu and Zn have a common source and these two metals are indeed typically found together in antifouling paints, as previously mentioned. Sacrificial zinc anodes may also be a source of Zn to the water, but this source is believed to be minor compared to the release from antifouling paints (Ytreberg et al. 2010). A strong relationship between the two metals was not only found for the sediments but for all measurements (Fig. 8). There is however more scatter in the regression for the soil samples which is probably a result of the difference in chemical composition of the paint flakes discarded during hull maintenance as well as the inhomogeneous distribution of paint particles and dust on the ground. Cu and Zn leached from the hulls during anchorage are mixed in the water column before deposition in the sediments which may explain the more homogenous regression correlation there.

There is a tendency for a North to South gradient (from the service pier out to Pier A) in the surface sediments with concentrations increasing with increased proximity to the boatyard. It is possible that the sloping boatyard area that is located uphill from the marina is acting as a source of metals to the water. Metals in the soil may be leached with infiltrating rainwater and transported downhill to the marina with the groundwater. There could also be less mixing of the water further into the bay leading to a higher build-up of metals in the sediments there.

In 1992, the use of biocide-containing antifouling paints on boats anchored in freshwater lakes or the Gulf of Bothnia, i.e., waters with low fouling pressure, was prohibited (Swedish National Chemical Inspectorate 1993). Assuming that the sediments at 17 cm depth correspond to the year that the harbor was taken into use (1964) and a constant sedimentation rate, the year 1992 would correspond to a depth of 7.5 cm. Despite the implementation of the mentioned regulation, no decrease in Cu and Zn concentrations was visible in the sediments above 7.5 cm. On the contrary, concentrations have either remained constant or increased with decreased depth. It should however be noted that the mixing of surface sediments through the action of bioturbation, which typically occurs at depths <10 cm in freshwater systems (Reible 2013), could have affected the appearance of the upper parts of the profiles. Lack of awareness and the absence of inspections at the boat club to verify that boat owners are complying with the legislation is most likely the main reason for the potential continued use of biocide-containing antifouling paints at the site.

### Risk assessment, regulation, and recommendations

The high concentrations of Cu and Zn, and in some places even Pb, found in soil at both recreational boatyards may pose a risk to both human health (Pb) and the environment. These soil concentrations are not unexpected; several studies have previously shown that spent antifouling paint particles typically contain high concentrations of Cu and Zn (Singh and Turner 2009a; Turner 2010, 2014; Parks et al. 2010; Rees et al. 2014) and paint particles were clearly visible on the ground at both boatyards. Of further concern is the fact that the metals can be leached from the particles by rain water and through this process end up in the ground water (Jessop and Turner 2011). Paint particles may also be transported from the soil to the nearby aquatic environment where leaching of Cu and Zn will occur (Singh and Turner 2009b). The metals contained in paint particles that end up in the sediments, either indirectly through transport from land or directly through shedding from anchored boats, have been shown to become bioavailable upon ingestion by deposit feeders (Turner et al. 2008; Jones and Turner 2010).

The highest measured Cu, Zn, and Pb levels at site A were 226, 25, and 3 times higher than the LSL guideline value, respectively. For site B, the corresponding numbers were 37, 11, and 6. Regrettably, such high concentrations, or even higher, seem to be typical at boatyards all over Sweden (Eklund and Eklund 2014), but most boat owners are unaware of this fact. Of all three metals, the occasional very high levels of Pb (up to 2536 mg/kg) in certain parts of the boatyards are of special concern to human health. Pb is known to affect the nervous system, with children being particularly sensitive (WHO 2010).

As is the case for many leisure boatyards in Sweden, and also true for the ones studied here, the land used for boat storage is leased from a municipality. A recent report investigating the liability of non-profit associations, such as recreational boat clubs, found that the clubs are not only ultimately liable for remediation of contaminated soil but are also obligated to implement protective measures to prevent damage from ongoing activities (Langlet et al. 2014). Continuing with business as usual may therefore become an expensive option. A way to reduce the load of metals to the soil could, for example, be to introduce rules in the boat club by-laws requiring members to cover the ground during sand blasting and/or to use vacuum scrapers and to dispose of the resulting debris appropriately. Such rules should be coupled with regular inspections to ensure compliance.

For the studied boat clubs in particular, efforts should also be made to ensure that the club members are aware of and complying with current regulation regarding the use of antifouling paints in freshwater. As both boatyards are located by a freshwater lake, biocide-containing paints are not allowed to be applied to the hulls. The sediment results, along with the high Cu and Zn concentrations in the top surface soil, indicate however that this regulation is not being respected. Half of the 880,000 recreational boats in Sweden are anchored in inland waters (The Swedish Transport Agency 2010) and this study suggests that it is likely that their owners are still using biocide-containing antifouling paints despite the 20-year-old ban.

## Conclusions

A new application using a handheld FPXRF to measure metals on boat hulls as a tool for regulatory management was recently presented (Ytreberg et al. 2015). This study, carried out with the same instrument, demonstrates the versatility of the FPXRF for risk assessment of boatyards and harbors. The evaluation of the FPXRF performance here shows that it is suitable for direct in situ screening of Cu, Zn, and Pb in soil as well as ex situ measurements of dried sediments. With its major advantage of a short analysis time, the FPXRF made it possible to map the metal contamination at the two boatyards. The investigation revealed that the majority (>90 %) of the amounts of Cu and Zn in the surface soil were concentrated in the areas of the boatyards specifically used for boat maintenance. In other words, the high levels of Cu and Zn measured in soil at the two boatyards were clearly shown to be linked to the scraping, sanding, and flushing of boat hulls painted with metal-containing antifouling paints over unprotected soil. Furthermore, the measurements also showed that the level of metal pollution of the boat storage areas increases with the age of the operation. It is therefore important for boat clubs to both take action to prevent further contamination and, most importantly, to act to ensure that their members are complying with current regulations and forgo the use of biocide-containing antifouling paints, if they are anchored in freshwater.
